# Detection of Circulating Tumor Cells in Resectable Pancreatic Ductal Adenocarcinoma: A Prospective Evaluation as a Prognostic Marker

**DOI:** 10.3389/fonc.2020.616440

**Published:** 2021-02-18

**Authors:** Byeong Geun Song, Wooil Kwon, Hyemin Kim, Eun Mi Lee, Young Min Han, Hongbeom Kim, Yoonhyeong Byun, Kyung Bun Lee, Kwang Hyuck Lee, Kyu Taek Lee, Jong Kyun Lee, Jin-Young Jang, Joo Kyung Park

**Affiliations:** ^1^ Department of Medicine, Samsung Medical Center, Sungkyunkwan University School of Medicine, Seoul, South Korea; ^2^ Departments of Surgery, Seoul National University College of Medicine, Seoul, South Korea; ^3^ Medical Research Institute, Sungkyunkwan University School of Medicine, Seoul, South Korea; ^4^ Departments of Pathology, Seoul National University College of Medicine, Seoul, South Korea; ^5^ Department of Health Sciences and Technology, SAIHST, Sungkyunkwan University, Seoul, South Korea

**Keywords:** circulating tumor cell, plectin-1, epithelial cell adhesion molecule, pancreatic ductal adenocarcinoma, overall survival

## Abstract

Circulating tumor cells (CTCs) are useful biomarkers of many solid tumors, but are infrequently detected in early stage pancreatic ductal adenocarcinomas (PDACs). The first drainage of pancreatic venous blood flow come to portal vein and pass through the liver, and they finally go out for peripheral blood. We thought that comparing CTCs from portal vein and peripheral blood could enable us to understand the clinical meaning of CTCs from each different site in PDACs. Therefore, we aimed to determine 1) whether CTCs could be reliably identified in early stages (operable) of PDACs, 2) if there are any differences in the detected number of CTC in portal vein blood and peripheral blood, and 3) whether CTCs can be sensitive biomarkers for the prognosis of resectable PDAC patients. Newly diagnosed PDAC patients who underwent operation with curative intention between 2013 and 2015 were prospectively enrolled. Blood draws from portal and peripheral vein ran through the microfabricated porous filter, and anti-epithelial cell adhesion molecule (EpCAM) and anti-Plectin-1 antibodies were used for CTC identification. Baseline clinical characteristics, tumor characteristics, treatment, and clinical outcomes were assessed. The clinical stages of the 32 enrolled patients were as follows: IA/IB 1 (3.1%); IIA 9 (28.1%); IIB 17 (53.1%); III 5 (15.6%). Twenty-seven patients (84.4%) received R0 resection, while five patients (15.6%) received R1 resection. EpCAM+ CTCs were detected in 20 portal blood (62.5%) and 22 peripheral blood (68.8%). Plectin-1+ CTCs were identified in 14 portal blood (43.8%) and 16 peripheral blood (50%). Plectin-1-expressing CTCs were picked from CTC platform (microfabricated porous filter) and we could find out all KRAS mutation. Patients with detectable EpCAM+ CTC less than one in peripheral blood showed longer overall survival (OS) compared to patients with detectable CTCs more than one (35.5 months vs. 16.0 months). EpCAM and Plectin-1 successfully identified CTCs at the early stage of PDACs. Also, the number of CTCs could be a prognostic marker for survival in resectable PDACs.

## Introduction

Pancreatic ductal adenocarcinoma (PDAC) is associated with poor prognosis due to early metastatic spread. The 5-year survival rate for metastatic PDAC is approximately 2% (1). Only 20% of patients have resectable PDAC at diagnosis, and only 20% of them survive for more than 5 years (2). Nonetheless, early diagnosis and curative resection is the only means to improve prognosis of PDAC patients. However, there are no useful biomarkers for early diagnosis, and predicting prognosis and treatment response. Among several novel biomarker candidates, circulating tumor cell (CTC) is one of the most promising candidates. Capturing, isolating, and characterizing CTCs have been developed, which revealed that CTCs usually circulate in the blood of patients with various cancers such as breast, lung, prostate and colorectal cancers (3–10). Several studies have been reported the prognostic and predictive value of CTCs in cancer patients (11–14). It is easy to detect CTCs in the peripheral circulation of cancer patients where tumor drainages are into the peripheral circulation. However, the first venous drainage of PDAC is into the portal circulation, leading some difficulties to capture CTCs in peripheral circulation. Thus, portal vein could be a more promising location for the detection of CTCs in PDAC. The investigation of CTCs in portal vs. peripheral blood may give us insights into the importance of tumor drainage pattern in CTC detection.

Antibodies against epithelial cell adhesion molecule (EpCAM) have been known to provide the specificity for CTC capture from blood because EpCAM is often overexpressed by epithelial tumors including breast, prostate, colon, and lung cancers (15). Plectin-1 is a cytolinker protein and was recently suggested as a biomarker for PDAC (16). As it was identified not only in primary and metastatic PDAC but also in pre-invasive Pancreatic Intraepithelial Neoplasia (PanIN) III lesions, Plectin-1 may be detected in early stage PDAC. It can also distinguish malignant pancreatic disease from chronic pancreatitis (16). In conclusion, Plectin-1 might be an ideal biomarker for PDAC. However, till date, it has not been studied to verify the usefulness of CTC detected by Plectin-1.

This study is the first to explore several aspects of CTCs in resectable PDACs. Firstly, the reliability of a microfabricated porous filter in identifying CTCs in patients with resectable PDACs was tested. Secondly, the feasibility of Plectin-1 as PDAC-specific identifier was examined. Thirdly, the clinical significance of CTCs captured from the portal vein was compared with that of CTCs captured from peripheral circulation. Lastly, the prognostic significance of number of CTCs was evaluated in terms of overall survival (OS).

## Patients and Methods

### Study Design and Blood Draws

Thirty-two newly diagnosed PDAC patients who underwent surgery with curative intention between 2013 and 2015 at Seoul National University Hospital were prospectively enrolled. The study patients were followed up until February of 2020 (5 years after the last study patient). Intraoperatively, 10 ml of blood was drawn from the portal vein from the surgical field just before complete resection and extraction of specimen. Simultaneous, 10 ml of blood was drawn from the cephalic vein. The blood samples were collected in Ethylenediaminetetraacetic acid (EDTA) tubes and processed within 4 h. Clinical variables collected included: age at diagnosis, sex, stages defined by the eighth edition American Joint Committee on Cancer (AJCC), date of surgery, date and site of recurrence, date of death and laboratory data. This study was conducted under the principles of the Declaration of Helsinki. The institutional review board (IRB) of Seoul National University Hospital approved the study protocol. All patients provided written informed consent, and all specimens were collected according to IRB regulations and approval (IRB No. 1305-573-489).

### Flow Cytometry Analysis and Immunofluorescent Staining

Pancreatic cancer cell lines were grown as a confluent monolayer in various culture media including Dulbecco’s Modified Eagle’s Medium for PANC1, SW1990, MIA-PaCa2, PaTu8902 and CFPAC-1 cells, RPMI-1640 Medium for COLO357, AsPC-1, KP-1NL, MPanc-96, BxPC3, PaTu8988, and CAPAN-2 cells, Iscove’s Modified Dulbecco’s Medium for CAPAN-1 cells, and Eagle’s Minimum Essential Medium for HPAFII at 37°C in a humidified atmosphere containing 5% CO2. All culture media were supplemented with 10% fetal bovine serum (FBS) and 1% Antibiotic Antimicotic, and they were purchased from Thermo Fisher Scientific (Waltham, MA, USA). After detaching with 0.25% Typsin/EDTA solution and washing with phosphate buffered saline (PBS), harvested cells were resuspended in ice cold flow cytometry staining buffer (PBS with 1% bovine serum albumin (BSA) and 0.1% sodium azide) to a concentration of 1x10^7^ cells/ml. Primary EpCAM or Plectin-1 antibody (Cell Signaling Technology (CST), Danvers, MA, USA) was added into polystyrene round bottom tube containing cells (1x10^6^ cells/0.1 ml/tube) and cells were incubated for 60 min in dark on ice with gentle shaking. After washing three times, cells were incubated with fluorochrome (APC for EpCAM, Alexa 594 for Plectin-1)-conjugated secondary antibody (CTS) for 30 min. Followed by rinsing cells, resuspended cells were analyzed on FACS Calibur flow cytometry system (BD Biosciences, San Jose, CA, USA). For immunofluorescent (IF) staining, pancreatic cancer cell lines were seeded on coated glasses, and they were stained with Alexa488-conjugated EpCAM, Plectin-1 (CST) and Alexa594-conjugated CD45 (eBioscience, San Diego, CA, USA) for 2 h at room temperature. After additional staining with Alexa488-conjugated anti-rabbit IgG (CST) for Plectin-1, all nuclei were counterstained with 4’6-diamidino-2-phenylindole (DAPI). Stained cells were observed under the Eclipse Ti2 inverted microscope (Nikon, Tokyo, Japan) with 10x and 20x objectives.

### Microfabricated Filter-Based CTC Enrichment

CTCs were enriched from 10 ml whole blood samples using a microfabricated porous filter according to the manufacturer’s instruction (Cytogen Inc., Seoul, Korea) (17). The Buffy coat was separated by Ficoll-Hypaque density gradient centrifugation, and passed through the nickel electroformed membrane filter designed with the size of the square pores to 6.5×6.5 μm and gap size of 6 μm. The resulting CTCs were recovered and processed for IF staining.

### CTC Immunostaining and Enumeration

For CTC enumeration, recovered cells were fixed with 4% formaldehyde for 10 min at room temperature and permeabilized for 15 min with 0.2% Triton X-100 in PBS. After blocking with 1% BSA in PBS for 30 min, they were incubated with primary antibodies against EpCAM, Plectin-1 (CST), and CD45 (eBioscience). This was followed by matched fluorochrome (Alexa 488 for EpCAM, Alexa 647 for Plectin-1, Alexa 594 for CD45)-conjugated secondary antibody incubation. Nuclei were counterstained with DAPI. CTCs were identified on the basis of cell size, morphology, and fluorescence staining based on EpCAM or Plectin-1 expression. Total cells were counted by DAPI staining, white blood cells (WBCs) were identified by CD45 staining. Captured cells were determined to be CTC if they were CD45 negative and EpCAM or Plectin-1 positive. Then number of CTCs/ml was determined by comprehensive image analysis. A count of one or more CTCs per ml of blood was defined as positive.

### Single Cell Isolation and KRAS Mutation Analysis

After CTC enrichment from the blood of PDAC patients, CTCs were stained with Plectin-1 antibody and DAPI. Two Plectin-1^+^ CTCs were isolated in a tube using CellCelector (ALS Automated Lab Solutions GmbH, Jena, Germany), and whole genome amplification (WGA) was carried out by using REPLI-g single cell kit (Qiagen, Venlo, Netherlands). Droplet Digital polymerase chain reaction (ddPCR) was performed using 2X ddPCR Supermix (Bio-Rad, Hercules, CA, USA) for KRAS probes (KRAS wild-type (WT) 5’-HEX-AGTTGGAGCTGGTGGCGTA-BHQ1-3’; KRAS mutant G12D 5’-FAM-AGTTGGAGCTGATGGCGTAG-BHQ1-3’; KRAS mutant G12V 5’-FAM-AGTTGGAGCTGTTGGCGTAG-BHQ1-3’) by QX200 Droplet Digital PCR System (Bio-Rad). After Plectin-1^+^ cell isolation, remaining cells captured on the filter membrane were also processed to WGA and ddPCR for KRAS WT and G12D mutant. Data analyses were performed as recommended by the manufacturer using the QuantaSoft Software version 1.7.4. (Bio-Rad).

### Statistical Analysis

Statistical significance of difference among continuous variables and categorical variables was examined using Student’s t-test (or Mann-Whitney’s test, as appropriate) and Chi-square test (or Fisher’s exact test, as appropriate), respectively. OS was calculated and plotted using Kaplan-Meier’s method and compared by log-rank test. We compared for the number of CTCs between-patients and within-patients for portal vein vs. peripheral vein, p-value<0.05 was considered statistically significant. Statistical analysis was performed using SPSS version 23.0 (SPSS Inc., Chicago, IL) and GraphPad Prism8.0 (GraphPad Software Inc., LA Jolla, CA).

## Results

### Evaluation of EpCAM and Plectin-1 Expression in PDAC Cells

**Figure 1:**
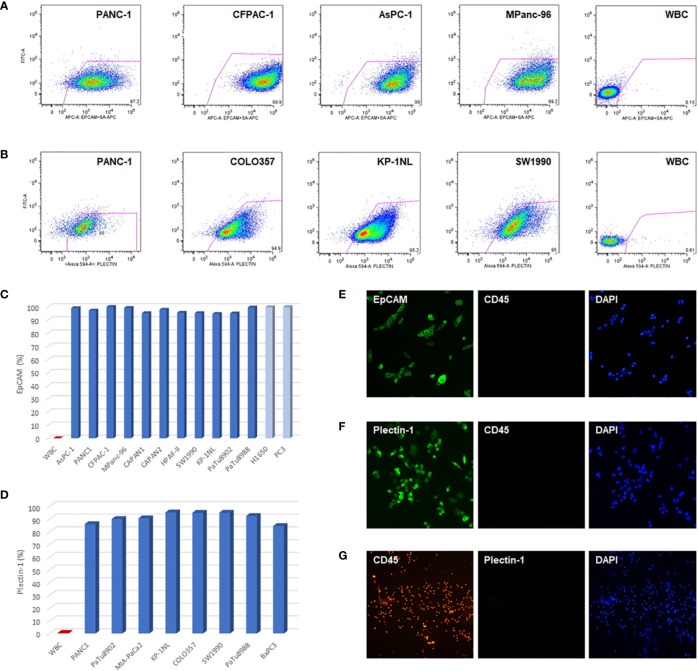
EpCAM and Plectin-1 expression in PDAC cells. To evaluate the anti-EpCAM and anti-Plectin-1 antibodies for detecting CTCs, the expression of EpCAM and Plectin-1 antigen was examined in PDAC cell lines by flow cytometry. **(A)** The surface expression of EpCAM in PDAC cell lines (PANC-1, CFPAC-1, AsPC-1, MPanc-96). **(B)** The expression of Plectin-1 in PDAC cell lines (PANC-1, COLO357, KP-1NL, SW1990). The expression of **(C)** EpCAM and **(D)** Plectin-1 in various PDAC cell lines were represented as percentages. WBCs were used as negative controls. To examine the expression of EpCAM and Plectin-1, **(E)** CAPAN-1 cells were stained with EpCAM (green) and CD45 (red), **(F)** MIA-PaCa2 cells were stained with Plectin-1 (green) and CD45 (red), and **(G)** WBCs were stained with CD45 (red) and Plectin-1 (green). All nucleated cells were stained with DAPI (blue).

To evaluate the feasibility of EpCAM and Plectin-1 antibodies in verifying CTCs of PDAC patients, the expressions of EpCAM and Plectin-1 were examined in human PDAC cell lines by flow cytometry. Most cells expressed EpCAMs in PDAC cells including PANC-1, CFPAC-1, AsPC-1, CAPAN-1, CAPAN-2, HPAFII, SW1990, KP-1NL, PaTu8902, PaTu8988, and MPanc-96 (**Figures 1A, C** **)**, which was compatible with other cancer cell lines such as prostate cancer cells (PC3) and non-small cell lung cancer cells (H1650). In addition, IF staining showed that most CAPAN-1 cells intensely expressed EpCAM (**Figure 1E**). The expression of Plectin-1 was also abundant in PDAC cells such as PANC-1, COLO357, KP-1NL, PaTu8902, PaTu8988, MiaPaCa2, BxPC3, and SW1990 (**Figures 1B, D** **)**. Otherwise, WBCs that could be contaminated during CTC preparation rarely expressed EpCAM or Plectin-1 on their surfaces (<1%). In addition, Plectin-1 was extensively expressed in MIA-PaCa2 cells (**Figure 1F**), but not in WBCs (**Figure 1G**). Therefore, it seems that EpCAM and Plectin-1 are suitable for identifying CTCs in PDACs.

### Detection of KRAS Mutation in Plectin-1-Expressing CTCs

**Figure 2:**
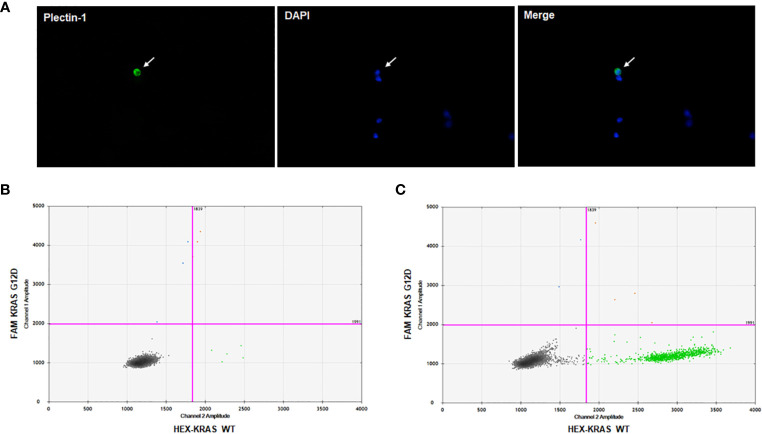
Plectin-1^+^ cell isolation and KRAS mutation analysis. To clarify Plectin-1-expressing cells in the blood, immunofluorescence staining was carried out to isolate Plectin-1-expressing cells, and ddPCR for KRAS wildtype (WT) and mutant (G12D) was performed. **(A)** A representative image of the circulating tumor cell (CTC) stained with Plectin-1 antibody (green) and 4’6-diamidino-2-phenylindole (DAPI) (blue). Arrow indicates the Plectin-1^+^ CTC. The detection of KRAS G12D mutation in **(B)** Plectin-1^+^ CTCs and **(C)** all capture cells on the microfabricated porous filter in the same patient.

To prove circulating cells expressing Plectin-1 in the blood sample from PDAC patients were CTCs of PDAC, we performed singe cell isolation and ddPCR for KRAS mutations. After CTC enrichment with microfabricated porous filter, CTCs on the filter membrane were stained with Plectin-1 and DAPI (**Figure 2A**). Then, Plectin-1^+^ CTC was picked under fluorescent microscopy, and a small amount of DNA was amplified to process to the ddPCR for KRAS wild-type (WT) and KRAS mutant (G12D). As a result, the Plectin-1^+^ CTC had KRAS G12D mutation (**Figure 2B**), and KRAS G12D mutation was also detected in bulk captured cells on the membrane (**Figure 2C**). It indicates that circulating Plectin-1^+^ cells in the blood of PDAC patient are circulating tumor cells of PDAC.

### Optimization and Validation of Plectin-1 Identification Antibody in Healthy Controls

**Figure 3:**
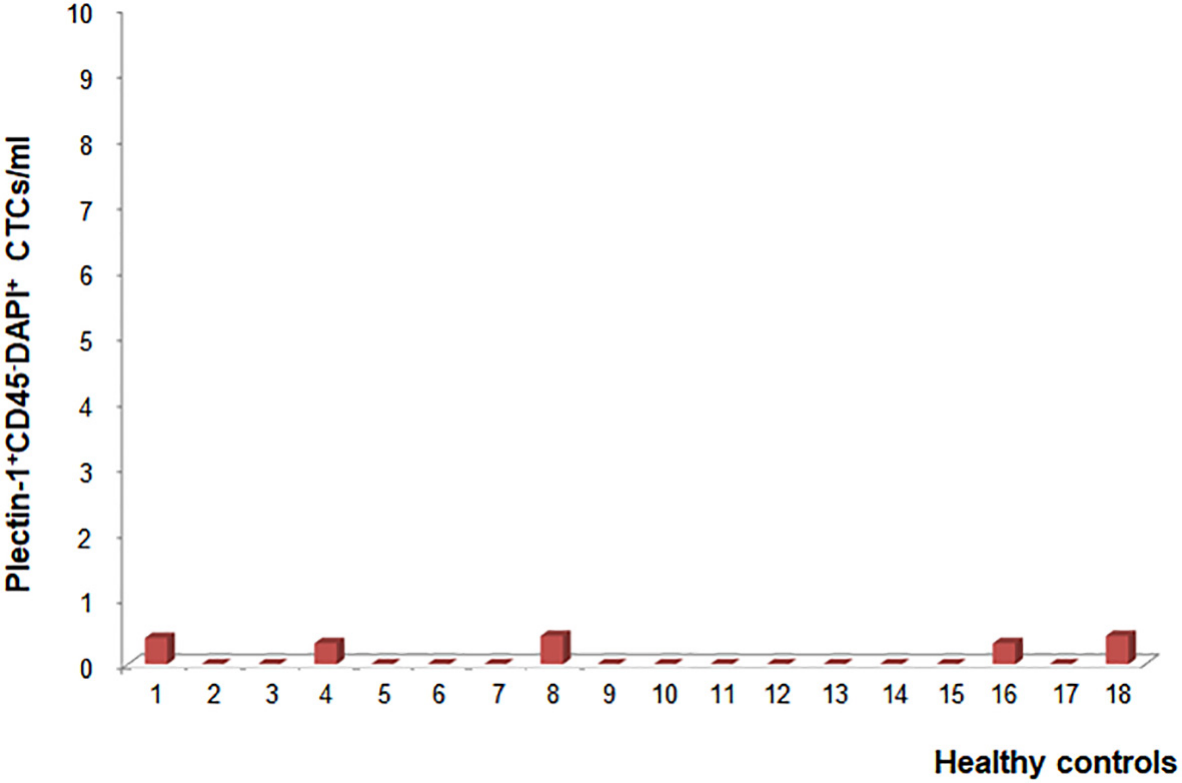
Optimization and validation of Plectin-1 in healthy controls. To validate the specificity of Plectin-1 antigen in detecting circulating tumor cells (CTCs), the number of CTCs was counted in peripheral blood samples of eighteen healthy volunteers after immunostaining with anti-Plectin-1 and anti-CD45 antibody. Plectin-1^+^CD45^-^DAPI^+^ CTC counts per 1 ml blood were calculated individually.

We examined the circulating Plectin-1^+^ cells in healthy controls to validate the specificity of Plectin-1 antibody. Peripheral blood was collected from 18 healthy volunteers, CTCs were enriched and processed for immunostaining with anti-Plectin-1 and anti-CD45 antibodies. Five samples showed some of positive staining of Plectin-1 antibody and the number of Plectin-1+ CTC was only 0.3–0.4 per 1 ml blood sample of healthy controls (Mean ± SD, 0.09 ± 0.16 CTCs/ml; Median 0.00 CTCs/ml). The rest of 13 samples showed no positive staining of Plectin-1 antibody (**Figure 3**). We have defined one or more CTCs per 1 ml of blood is positive for CTC capturing and therefore, there was no positive CTCs with Plectin-1 antibody in healthy volunteers.

### Identification of CTCs in PDAC Patients

**Figure 4:**
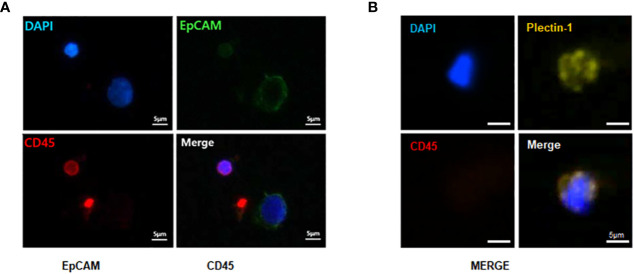
Immunofluorescence staining of captured circulating tumor cells (CTCs) from patients with resectable pancreatic ductal adenocarcinomas (PDACs). To identify CTCs, captured CTCs from PDAC patients were immunostained with anti-epithelial cell adhesion molecule (EpCAM) (green), anti-Plectin-1 (gold) and anti-CD45 (red) antibodies. Nuclei were counterstained with DAPI (blue). CTCs are **(A)** EpCAM^+^ or **(B)** Plectin-1^+^ among CD45^-^DAPI^+^ cells. Scale bar, 5 μm.

The identification of CTCs in PDACs was based on immunostaining with anti-EpCAM and anti-Plectin-1 antibodies. All nucleated cells were verified with DAPI staining, and WBCs were excluded with CD45 staining. We defined DAPI^+^CD45^-^EpCAM^+^ (**Figure 4A**) or DAPI^+^CD45^-^Plectin-1^+^ cells (**Figure 4B**) as PDAC CTCs. It indicated that CTCs are reliably identified in the blood at the early stage of PDACs.

### Baseline Characteristics and Clinical Course of Study Patients

Baseline characteristics of 32 study patients are summarized in **Table 1**. Twenty-one male and eleven female patients aged 44–81 years (median 62 years). Distribution of stage was as follows: stage IA/IB 1 (3.1%); stage IIA 9 (28.1%); stage IIB 17 (53.1%); stage III 5 (15.6%). Of all the patients, 22 (68.8%) received pancreaticoduodenectomy, nine (28.1%) received distal pancreatectomy, and one (3.1%) received total pancreatectomy. Twenty-seven patients (84.4%) received R0 resection, while five patients (15.6%) received R1 resection. Twenty-six patients (81.3%) received adjuvant treatment, and five patients (15.6%) received neoadjuvant treatment. Median (range) of total bilirubin, Carbohydrate Antigen (CA) 19-9, and Carcinoembryonic Antigen (CEA) were 1.0 mg/dl (0.2–19.2), 156.6 IU/ml (2.0–12,000), and 2.4 ng/ml (0.7–51.2), respectively.

**Table 1:** Patient characteristics (n = 32).

Characteristics	n = 32
Age, median (range)	62 (44–81)
Sex, n (%)	
Male	21 (65.6)
Female	11 (34.4)
Stage (pAJCC)*, n (%)	
IA, IB	1 (3.1)
IIA	9 (28.1)
IIB	17 (53.1)
III	5 (15.6)
Name of Operation, n (%)	
Pancreatoduodenectomy	22 (68.8)
Distal pancreatectomy	9 (28.1)
Total pancreatectomy	1 (3.1)
Resection margin, n (%)	
R0	25 (78.1)
R1	7 (21.9)
Adjuvant treatment, n (%)	28 (87.5)
Neoadjuvant treatment, n (%)	6 (18.8)
Total bilirubin, median (range)	1.0 (0.2–19.2)
CA 19-9 (IU/ml), median (range)	156.6 (2.0–12,000)
CEA (ng/ml), median (range)	2.4 (0.7–51.2)

CA 19-9, carbohydrate antigen 19-9; CEA, carcinoembryonic antigen.

*pathological stage according to the AJCC.

The study patients were followed up until February of 2020 (5 years after the last study patient). The median follow-up period of 32 patients was 19 months (range: 4 – 70). The median progression free and overall survivals were 11 (range: 0 – 68) and 19 months (range: 4–70), respectively. Recurrence was found in 27 patients (84.4%), of which only two were locoregional and 25 involved systemic recurrence (six local and systemic, and 19 systemic). The most common site of distant metastasis was the liver (n=10) followed by peritoneal seeding (n=6) and lung (n=5). Twenty-seven patients were dead at last follow-ups. Among five survivors, four were free of disease and one was alive with lung metastasis.

### Comparison of the Number of CTCs Detected in Portal Draws and Peripheral Blood

**Figure 5:**
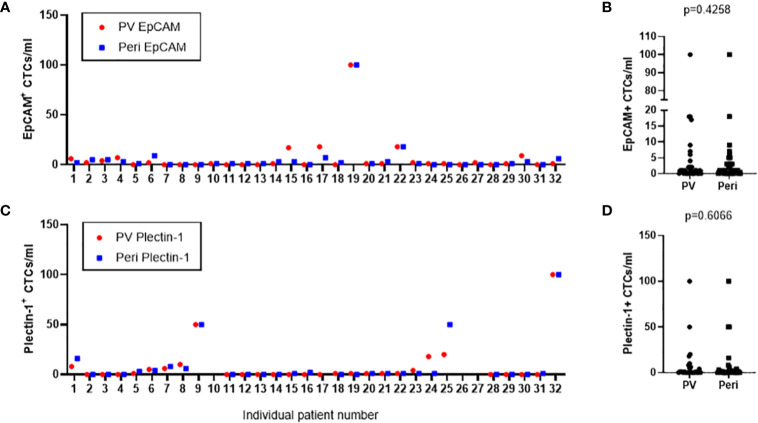
Number of circulating tumor cells (CTCs) detected from the study patients in both portal vein and peripheral blood draws. CTCs were isolated from portal vein (PV) and peripheral vein (Peri) of 32 PDAC patients. **(A)** EpCAM^+^ CTCs in PV and Peri blood were enumerated from each patient. **(C)** Plectin-1^+^ CTCs in PV and Peri blood were counted from each patient. **(B)** CTCs positive to epithelial cell adhesion molecule (EpCAM) or **(D)** CTCs positive to Plectin-1 were compared in PV and Peri blood samples.

CTCs were enriched and enumerated from both portal draws and peripheral blood of 32 resectable PDAC patients. As a result, EpCAM^+^ CTCs were detected in 20 (62.5%) portal blood and 22 (68.8%) peripheral blood samples, ranged from 0 to 100 in portal blood and peripheral blood (**Figure 5A**). There was no significance in the number of EpCAM^+^ CTCs between portal and peripheral bloods (p=0.426) (**Figure 5B** **)**. Plectin-1^+^ CTCs were identified in 14 (43.8%) portal and 16 (50.0%) peripheral blood samples. Captured CTCs with anti-Plectin-1 antibody ranged from 0 to 100 in portal vein and in peripheral vein (**Figure 5C** **)**. There was also no significant difference in the number of CTCs detected with Plectin-1 antibody between peripheral vein and portal vein (p=0.607) (**Figure 5D** **)**. In addition, there was no significant difference of CTC numbers between patients with R0 resection and patients with R1 resection (p = 0.387) (**Supplementary Table 1**).

### CTC Enumeration for Staging

**Figure 6:**
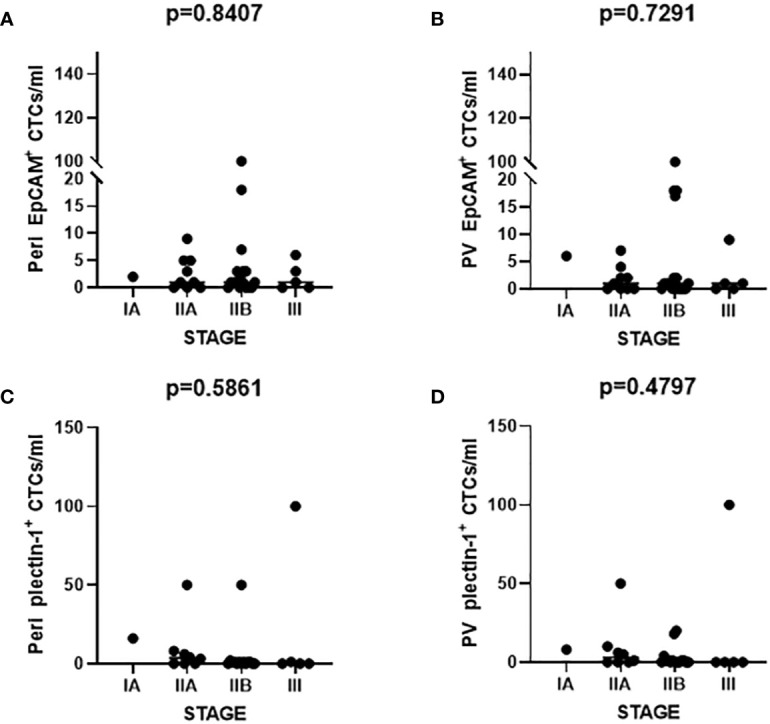
Number of circulating tumor cells (CTCs) according to the American Joint Committee on Cancer (AJCC) staging. The number of CTCs identified with epithelial cell adhesion molecule (EpCAM) from **(A)** peripheral vein (Peri) and **(B)** portal vein (PV) was enumerated. CTCs detected from **(C)** Peri and **(D)** PV were counted after Plectin-1 immunostaining. Each count was evaluated according to the AJCC staging.

The number of captured CTCs using EpCAM and Plectin-1 was analyzed according to the 8^th^ AJCC staging system. There was no significant difference in the number of CTCs detected across stages with EpCAM from peripheral vein, p=0.841; EpCAM from portal vein, p=0.729; Plectin-1 from peripheral vein, p=0.586; Plectin-1 from portal vein, p=0.480 (**Figure 6** **).** It seems that the number of CTCs is not a predictive marker for staging in resectable PDAC patients.

### Survival Analysis According to the Number of CTCs

**Figure 7:**
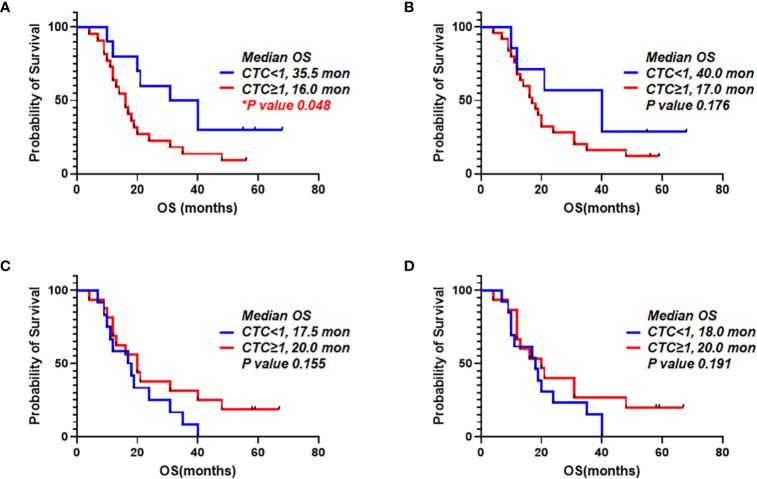
Overall survival correlated to number of circulating tumor cells (CTCs) detected using epithelial cell adhesion molecule (EpCAM) and Plectin-1 in peripheral blood and portal blood. CTC counts based on EpCAM and Plectin-1 expression were evaluated with Overall survival (OS) of 32 PDAC patients. Patients were divided into two groups depending on detectable EpCAM^+^ CTCs in **(A)** Peripheral vein and **(B)** Portal vein. Patients were divided into two groups depending on detectable Plectin-1^+^ CTCs in **(C)** Peripheral vein and **(D)** Portal vein. OS was analyzed with the number of CTCs using Kaplan-Meier estimator.

It was examined that the prognostic value of CTCs from portal vein and peripheral blood. We compared patients with one or more CTCs vs. less than one CTC. When we detected CTCs using EpCAM in peripheral blood, OS for patients with one or more CTCs and patients with CTC under one were 16.0 and 35.5 months, respectively (p=0.048). PDAC patients having EpCAM^+^ CTCs in peripheral circulation showed significantly worse prognosis (**Figure 7A** **)**. For detection of EpCAM^+^ CTCs in portal vein, OS for patients with one or more CTCs and CTC under one were 17.0 and 40.0 months, respectively (p=0.176) (**Figure 7B** **)**. In addition, OS for patients with more than one CTCs using Plectin-1 in peripheral blood and OS for patients less than one CTC were 20.0 and 17.5 months, respectively (p=0.155) (**Figure 7C**). For detection of CTCs in the portal blood using Plectin-1, OS for patients with more than one CTCs and less than one CTC were 20.0 and 18.0 months, respectively (p=0.191) (**Figure 7D**). For both portal and peripheral bloods, CTC enumeration based on Plectin-1 expression could not predict the survival of PDAC. However, peripheral EpCAM^+^ CTCs could be a prognostic marker for resectable PDACs.

## Discussion

In this study, CTCs were reliably captured by a microfabricated porous filter from resectable patients at the early stage of PDAC. We verified Plectin-1 as a practical PDAC CTC identifier. CTCs from peripheral circulation and portal drainage had no remarkable differences in the number, and only EpCAM^+^ CTCs in peripheral blood was a factor associated with prognosis whereas others were not.

PDAC is fatal because of its early metastasis (18, 19). Thus, early findings of subclinical metastasis are clinically significant in diagnosis. In this regard, CTCs are good candidates for detection of early metastasis. However, the association between prognosis and CTCs in resectable PDAC patients had not been studied elaborately. Moreover, there are no practical methods to identify PDAC-specific CTCs.

Mehmet et al. first developed a unique microfluidic platform (CTC-chip) capable of efficient and selective separation of viable CTCs from peripheral whole bloods. It was mediated by the interaction of target CTCs with EpCAM antibody-coated microspots under precisely controlled laminar flow conditions, without requisite pre-labeling or processing of samples (20). Although the method of using EpCAM as a capture antibody is now widely used, EpCAM-based enrichment systems are associated with the problem that EpCAM expression of CTCs might be down-regulated during the epithelial mesenchymal transition. CTC isolation dependent on epithelial markers such as EpCAM and cytokeratin might miss mesenchymal type CTCs. Label-independent enrichment methods including size-based selection, inertial focusing-based selection, dielectric field-based selection, and density-based approaches were also developed. Additionally, a combination of different enrichment strategies has been practically used (21, 22). We utilized a size-based microfabricated porous filter platform and label-dependent detection for CTCs. Moreover, several studies have tried to detect heterogenous CTCs by using additional markers, for example, mesenchymal markers including vimentin, N-cadherin, and twist1, and stemness markers such as CD133, CD44, and ALDH1 (23). There have been efforts to find a better marker for identifying CTCs specifically for PDAC. Recently, Plectin-1 was suggested as a biomarker for PDAC, and broadly expressed from pre-invasive lesions to metastatic PDACs (16). Also, it was verified that circulating cells expressing Plectin-1 in the blood of patients with PDACs had KRAS G12D mutation (**Figure 2B** **)**. A very high rate of activating mutations in KRAS (>90%) is included in PDAC (24), and KRAS G12D is the most dominant one (25). In this sense, Plectin-1 is a fascinating target for CTC platform development. This study is the first report to examine the feasibility of Plectin-1 as a new detection marker for PDAC CTCs, and showed that Plectin-1 can be reliably used to detect CTCs in resectable PDAC patients. Plectin-1^+^ CTC counts were not significantly different from EpCAM^+^ CTC counts in both portal vein and peripheral vein (**Figure 5**). Further investigations are needed to clarify whether Plectin-1 was co-expressed in EpCAM^+^ CTCs and whether captured CTCs expressed one of two.

The number and detection rate of CTCs were higher in portal vein compared to peripheral blood in pancreatobiliary cancer and colorectal cancer (26, 27). However, the count and frequency of EpCAM^+^ or Plectin-1+ CTCs were not statically significant between portal and peripheral vein (**Figure 5**). Furthermore, CTC counts utilizing microfluidic NanoVelcro CTC chips were correlated with PDAC stage (28), which was contradictory to our result that numbers of EpCAM^+^ and Plectin-1^+^ CTCs were not dependent on the AJCC staging in resectable PDAC patients (**Figure 6** **)**. CTC-positivity was not associated with tumor characteristics, lymph node metastasis, resectability, or advanced TNM stage (29), and the percentage of CTC detection was not related to the TNM stage or distant metastasis (30, 31). It should be considered that we utilized a size-based filter platform instead of an immunomagnetic platform, and the enrolled patients had resectable PDACs with prominently stage II but not advanced or metastatic PDAC.

The role of CTC is still controversial but many reports have shown promising results of CTCs as a tool for refining prognosis and identifying personalized treatment to patients in various gastrointestinal cancers including colorectal cancer and gastric cancer (32–34). Some studies have shown that CTCs can predict the survival rate, diagnosis, and stage of PDAC. CTC presence evaluated by using the CellSearch platform was an independent prognostic factor with respect to OS of advanced PDAC patients (35). Another study used size-based ScreenCell Cyto filtration device and hematoxylin-eosin-safran staining to detect CTCs, reported no differences in survival between patients with positive and negative results for CTC (36). Khoja et al. isolated CTCs by size and identified them solely by morphological characters such as nuclear to cytoplasmic ratio, diameter, hyperchromatic nuclei and cellular shape. They detected CTCs in 93% of pancreatic cancer patients, but did not find any significant difference in either regular survival or progression-free survival (37). The debatable conclusion seems to be associated with varied detection and identification methods.

To date, there are only few studies investigating the prognostic relevance of CTCs in both PV and peripheral blood in PDAC. There were some studies reporting that CTC from portal vein predicts liver metastasis in resectable PDAC patients (38, 39), but no reports have explicitly investigated the correlation between survival rate and CTCs in portal draws as well as peripheral blood of patients with resectable PDAC. We enrolled PDAC patients from 2013 to 2015 prospectively, and followed up till the February of 2020 to collect the complete survival data. In **Figure 7**, EpCAM^+^ CTC counts in peripheral blood was adversely associated with OS, and more than one EpCAM^+^ CTCs in portal vein was not significantly related to OS. The enumeration of Plectin-1^+^ CTCs in both peripheral blood and portal vein showed none of significant association with OS. In addition, overall recurrence did not differ between the patients without portal vein EpCAM^+^ CTCs and those with portal vein EpCAM^+^ CTCs (83.3% vs. 85.0%, p > 0.999). Also, we did not find statistically significant difference between the patients without portal vein EpCAM^+^ CTCs and those with portal vein EpCAM^+^ CTCs (**Supplementary Table 2**).

We need more investigation of EpCAM^+^ and Plectin-1^+^ CTCs with larger groups of PDAC patients. Further insights into the character and genetic signature of CTCs compared to the parent tumor in surgical patients may allow us to detect or predict recurrence patterns, location and time of relapse.

Here, we would like to address the practicability of Plectin-1 to detect CTCs and the prognostic value of CTCs positive for EpCAM and Plectin-1 from both peripheral and portal venous blood of resectable PDAC patients. In conclusion, Plectin-1 is reliable in identifying CTCs captured by microfabricated filter, and the enumeration of EpCAM^+^ CTCs in peripheral blood might be associated with poor prognosis in resectable PDAC patients.

## Data Availability Statement

The raw data supporting the conclusions of this article will be made available by the authors, without undue reservation.

## Ethics Statement 

Ethical approval was obtained from the institutional review board of Seoul National University Hospital (IRB no. 1305-573-489). The patients/participants provided their written informed consent to participate in this study.

## Author Contributions

BGS, WK, HK: study concept, study design, data acquisition, data analysis, data interpretation, and drafting of the manuscript; EML, YMH, HK, YB: data acquisition; KBL: data interpretation; KHL, JKL, KTL: data acquisition and critical revision of the manuscript for important intellectual content; J-YJ and JKP: study concept, study design, data acquisition, and study supervision. All authors contributed to the article and approved the submitted version.

## Funding

This project was supported by intramural fund, Seoul National University Hospital Research Fund (grant 04-2013-0990) and Korean Gastroenterology Fund for Future Development. This material is based on the work supported by the Ministry of Trade, Industry & Energy (MOTIE, Korea) under Industrial Technology Innovation Program (grant10047614, Development of digital cell imaging system). This work was supported by a grant from SK Chemical Research Fund of the Korean Society of Gastroenterology (grant 800-20130378).

## Conflict of Interest

The authors declare that the research was conducted in the absence of any commercial or financial relationships that could be construed as a potential conflict of interest.
