# Supplementary material for: Detection of Circulating Tumor Cells in Resectable Pancreatic Ductal Adenocarcinoma: A Prospective Evaluation as a Prognostic Marker
Source: Front Oncol. 2021 Feb 18;10:616440. doi: 10.3389/fonc.2020.616440 (PMC7930477; doi:10.3389/fonc.2020.616440)
Supplement: Supplementary file 1 [file DataSheet_1.docx]

**Supplementary table 1. CTC numbers according to R0 and R1 resection in peripheral and portal bloods**

|  | R0 resection (n = 25) | R1 resection (n = 7) |
| --- | --- | --- |
| Peripheral EpCAM^+^ CTC negative | 9 (36.0%) | 1 (14.3%) |
| Peripheral EpCAM^+^ CTC positive | 16 (64.0%) | 6 (85.7%) |

**Supplementary table 2. Recurrence according to the portal vein circulating tumor cells**

|  | Total  (n=32) | PV EpCAM (-) (n=12) | PV EpCAM (+)  (n=20) | *P value* |
| --- | --- | --- | --- | --- |
| Recurrence free survival (months) | 20.3 ± 22.3 | 16.9 ± 22.9 | 22.4 ± 22.3 | 0.510 |
| Overall recurrence, n (%) | 27 (84.4) | 10 (83.3) | 17 (85.0) | >0.999 |
| Locoregional recurrence, n (%) | 8 (25.0) | 5 (41.7) | 3 (15.0) | 0.206 |
| Systemic recurrence, n (%) |  |  |  |  |
| Liver | 10 (31.2) | 5 (41.7) | 5 (25.0) | 0.555 |
| Lung | 5 (15.6) | 1 (8.3) | 4 (20.0) | 0.706 |
| Bone | 2 (6.2) | 1 (8.3) | 1 (5.0) | >0.999 |
| Peritoneal seeding | 6 (18.8) | 2 (16.7) | 4 (20.0) | >0.999 |
| Para-aortic lymph node | 2 (6.2) | 1 (8.3) | 1 (5.0) | >0.999 |
| Other systemic meta | 3 (9.4) | 0 (0.0) | 3 (15.0) | 0.434 |
